# Barriers and enablers to integrating maternal and child health services to antenatal care in low and middle income countries

**DOI:** 10.1111/1471-0528.13898

**Published:** 2016-02-09

**Authors:** TE de Jongh, I Gurol‐Urganci, E Allen, N Jiayue Zhu, R Atun

**Affiliations:** ^1^ Gephyra IHCAmsterdamthe Netherlands; ^2^ London School of Hygiene and Tropical MedicineLondonUK; ^3^ Department of Global Health and PopulationHarvard TH Chan School of Public HealthHarvard UniversityBostonMAUSA

**Keywords:** Antenatal care, integration, low and middle income countries

## Abstract

Antenatal care (ANC) represents a delivery platform for a broad range of health services; however, these opportunities are insufficiently utilised. This review explores key barriers and enablers for successful integration of health s"ervices with ANC in different contexts. Data from peer‐reviewed and grey literature were organised using the SURE checklist. We identified 46 reports focusing on integration of HIV, tuberculosis, malaria, syphilis or nutrition services with ANC from Asia, Africa and the Pacific. Perspectives of service users and providers, social and political factors, and health system characteristics (such as resource availability and organisational structures) affected ease of integration.

**Tweetable abstract:**

Health system factors, context and stakeholders must be considered for integrated antenatal care services.

## Tweetable abstract

Health system factors, context and stakeholders must be considered for integrated antenatal care services.

## Introduction

For most women in low‐ and middle‐income countries (LMIC), antenatal care (ANC) plays a highly important dual role: not only does ANC provide effective interventions to reduce the risks associated with pregnancy and childbirth, it can also serve as a delivery platform for other health services.^1^ Particularly in settings where the prevalence of HIV/AIDS, sexually transmitted infections (STIs), tuberculosis (TB) and malaria is high, integrating services for these conditions with ANC can significantly expand their reach.^2^ In fact, the World Health Organization (WHO) identified integration of ANC with other health programmes as a key strategy for reducing missed opportunities for patient contact and improving maternal and child health (MCH).^1^, ^3^, ^4^ Evidence from the countries studied, however, suggests that in practice integrated delivery of ANC with other health services is not systematic or adequate and that opportunities for providing care for women are lost.^5^, ^6^

Several factors enable or hinder the integration of other health services with ANC. There may be barriers in the specific health system context where services are delivered: for example, lack of trained health workers may prevent delivery of a full range of services, even if these are prescribed by national health policies. Factors related to broader political and social context can present significant hurdles for effective integration. Hence, delivery of integrated care that combines ANC with other health services to expand coverage and improve health outcomes requires an understanding of the main barriers and enablers to be successful.

We present a comprehensive review of drivers for integration of health programmes with ANC. The review combines published peer‐reviewed literature with grey literature sources. We provide a synthesis of the key barriers and enablers to integration in different contexts, bringing together randomised controlled trials and other published studies on how integrated delivery of ANC with other health services affects MCH outcomes, user experience, service access and coverage, and programme efficiency when compared with ‘routine’ models of care in which the same services were delivered separately.

## Methods

### Defining ‘integration’

For the purposes of this review, we considered any study that described a change from ‘routine practice’ with the intention of integrating provision of ANC services with other health services. Integrated service provision models include co‐location of services, using a single point of access; collaboration between different service providers involved in a woman's care (e.g. in integrated care teams) or a well‐organised referral system, with follow up and feedback among different service providers.

### Search strategy

The search for relevant studies included both electronic database searches for peer‐reviewed literature and conference proceedings as well as online searches for grey literature to identify qualitative evidence on the barriers and enablers for successful integration of health services with ANC.

The databases searched included the Cochrane Central Register of Controlled Trials (CENTRAL), Cochrane Database of Systematic Reviews (Cochrane Reviews), Cochrane Database of Abstracts of Reviews of Effects (Other Reviews), MEDLINE, EMBASE, CINAHL Plus, Global Health and POPLINE. We used a comprehensive search strategy with no language or publication date restrictions. The search string for MEDLINE, which was tailored to each of the databases, is provided in Appendix S1.

For grey literature, we searched clinical trial registers [ClinicalTrials.gov and Current Controlled Trials (ISRCTN)], electronic databases for grey literature [GreyNet International, The New York Academy of Medicine (NYAM) Grey Literature Report] and online directories and websites of organisations involved in MCH issues or global health. These included: WHO and WHOLIS; Maternal Health Task Force; Implementing Best Practice Initiative; World Bank; Count Down to 2015; Women Deliver; Catalyst Consortium; CEDPA; DFID; EngenderHealth; FHI; ICRW; INFO Project; IGWG; IPPF; IntraHealth International; Global Fund; JHPIEGO; JHUCCP; John Snow Inc.; Management Sciences for Health; Partners in Population and Development; PATH; Pathfinder International; PEPFAR; Pop Council; PSI; UNFAO; UNFPA and UNAIDS. The search string used in grey literature sources was ‘integrat* (e.g. integration, integrated, integrating) AND (maternal OR neonatal OR pregnancy OR mother OR infant or baby)’. There were no date restrictions. The electronic database search was completed in February 2014 and the grey literature search in March 2014.

### Selection and data extraction

This review followed a comprehensive selection process. In the first stage, the authors (T.dJ, E.A.) evaluated publications for their potential relevance based on titles. Any title judged as potentially relevant by either of the authors was next assessed for eligibility on the basis of the abstract. Due to the large number of abstracts, those on which the authors disagreed were independently reviewed by a third author (I.G.U.) who decided on its inclusion into the third round of screening, where two authors (T.dJ., E.A.) reviewed the full text of each retained publication and noted whether the study reported on an integrated service into ANC. In the final stage, one author (I.G.U.) screened all studies that focused on integration of other health services into ANC regardless of any study design considerations. Studies discussing any qualitative evidence on enablers and barriers for implementation were eligible for inclusion in this review. We extracted basic data from studies to a standardised form (including title, author, year of publication, country, setting, funding). We used the SURE checklist^7^ to facilitate data extraction on barriers and enablers, organise emerging findings and identify gaps in knowledge.^8^ The SURE checklist was developed to identify barriers to implementing health systems changes and has been used to evaluate the evidence on factors affecting implementation of large‐scale programmes aimed at scaling up human resources for maternal and newborn health.^8^ The factors are grouped in five main categories: (1) recipients of care, (2) providers of care, (3) other stakeholders, (4) health system constraints, and (5) social and political constraints), with potential barriers and enablers identified in each category.

## Results

### Search results

**Figure 1:**
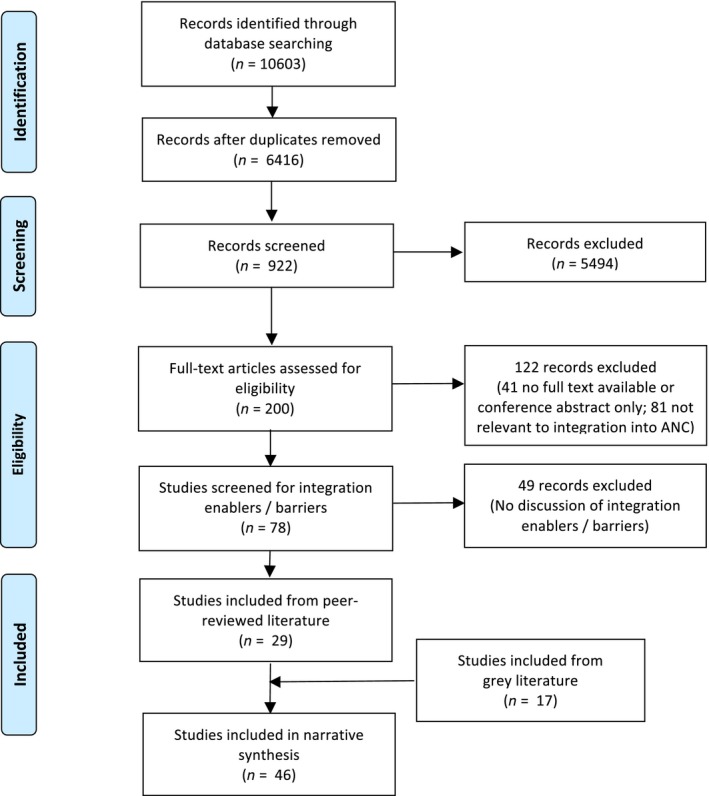
PRISMA flow diagram showing number of articles at each stage of the review.

Among 6416 unique citations retrieved from electronic databases, 922 titles were considered potentially relevant. Of these citations, 842 included abstracts that were subsequently reviewed. Among the abstracts, 120 were considered potentially relevant. For an additional 80 citations, no abstracts were available. These citations were all carried forward to the next stage of the screening process, in which the full text of the potentially eligible studies was reviewed. We retrieved the full text for 177 of 200 citations, among which 78 reported on ANC programmes involving integration of other health services. Only 29 of the 78 citations discussed implementation enablers and barriers to integration of services, and were considered eligible for inclusion in this review. A separate search of databases of grey literature and trial databases yielded 19 more potentially relevant reports from NYAM, three randomised controlled trials (RCTs) from ISRCTN and five RCT studies from ClinicalTrials.gov. None of these studies included information on enablers or barriers to integration of health services into ANC programmes. Thirteen relevant reports were identified from the websites of organisations involved in MCH issues and global health^9^, ^10^, ^11^, ^12^, ^13^, ^14^, ^15^, ^16^, ^17^, ^18^, ^19^, ^20^, ^21^, and four additional reports were identified through citation tracking.^22^, ^23^, ^24^, ^25^ In total, we thus identified 46 eligible reports (29 from peer‐reviewed and 17 from grey literature) for which we have here summarised the barriers and enablers to integration (Figure 1).

The included reports cover programmes from Africa (Botswana^26^, Cameroon^27^, Cote d'Ivoire^28^, ^29^, Ethiopia^30^, Kenya^18^, ^24^, ^31^, ^32^, ^33^, Malawi^30^, ^34^, Mozambique^35^, ^36^, Rwanda^10^, ^37^, South Africa^25^, ^38^, ^39^, ^40^, ^41^, ^42^, ^43^, Swaziland^44^, Tanzania^21^, ^23^, ^24^, ^45^, ^46^, Uganda^24^, ^47^, ^48^, Zambia^24^, ^37^, ^49^, Zimbabwe^50^ and Sub‐Saharan Africa in general^15^, ^16^), Asia (China^51^, Mongolia^52^, Thailand^53^), Europe (Ukraine^54^) and the Pacific (Fiji^13^). The majority of the emerging evidence relates to integration of HIV services, mainly prevention of mother‐to‐child transmission (PMTCT) of HIV^10^, ^11^, ^12^, ^13^, ^16^, ^17^, ^18^, ^19^, ^20^, ^21^, ^22^, ^23^, ^24^, ^25^, ^26^, ^27^, ^28^, ^29^, ^30^, ^31^, ^32^, ^33^, ^34^, ^35^, ^36^, ^38^, ^40^, ^42^, ^43^, ^44^, ^46^, ^47^, ^48^, ^50^, ^51^, ^53^, ^54^, one study is on the integration of PMTCT and TB services^39^, one on malaria^15^, five on syphilis^40^, ^42^, ^45^, ^49^, ^52^ and two on integration of nutrition services.^9^, ^20^ We also include guidance from the Global Fund to Fight AIDS, Tuberculosis and Malaria on how to integrate maternal, newborn and child health interventions with HIV, tuberculosis and malaria programmes.^14^

### Factors affecting integration of ANC with other health services

#### Recipients of care

Limited evidence from quantitative evaluations of integrated services suggests that integration can lead to improved uptake, earlier initiation of treatment and lower loss‐to‐follow up for women who require treatment.^21^, ^24^ These evaluations suggest that integrated programmes could provide an entry point for testing and counselling for other family members as well.^21^, ^24^ Participation of male partners has been low in PMTCT programmes, and innovative solutions for community mobilisation might be needed.^47^, ^50^ One specific strategy adopted in Fiji to incorporate partners into HIV care and reproductive health decision‐making was to distribute a ‘men's pack’ for all women in the antenatal programme, containing information, communication and education materials.^13^ In Cameroon, providers discussed culturally appropriate ways for mothers to present HIV test results to their spouses and strongly encouraged spouses to attend the first antenatal visit.^27^ Targeted invitation letters for partners were used in Zambia^49^ and Uganda.^48^ However, women may be reluctant to take up additional tests, particularly when their partners are involved. In South Africa, where TB screening is recommended as a routine component of ANC, the uptake has been lower than expected. A study by Kali et al.^16^, ^55^ suggests that pregnant women, particularly those recently diagnosed with HIV, were not willing to be screened for TB in order to avoid a positive diagnosis. Similarly, in Cote d'Ivoire, HIV‐infected women who lived with a partner showed poorer uptake of PMTCT.^29^ Couple‐counselling sessions, in particular those that stress the expected benefits for their children, could facilitate the uptake of PMTCT.^37^, ^48^

There is limited evidence on the perspectives and experiences of women and their partners of integrated ANC services. In a pilot programme of ART initiation in pregnancy in South Africa, most women appreciated rapid access to ART and found the provided counselling useful.^38^ HIV‐positive women in rural Kenya preferred an integrated ANC‐HIV model for its increased confidentiality (women were not readily identifiable as attending an HIV service), the ease of receiving comprehensive care in a single visit, and improved provider–patient relationships.^31^, ^32^ In Mongolia, patients preferred receiving syphilis testing in the same place as ANC, allowing them to get same‐day results and receive counselling and treatment from ANC providers.^52^ Similar results were reported in various other settings: coverage was lower for integrated services that were provided at different locations or those not provided on the same day, resulting in delayed testing and treatment initiation.^33^, ^34^, ^40^, ^41^, ^42^, ^45^

#### Providers of care

Training and clear care guidelines are essential for providing knowledge and skills to deliver integrated services. This training should be provided consistently to all health workers providing ANC. For integrated PMTCT, training in the recognition and staging of HIV infection can allow care and treatment of HIV‐infected pregnant women to occur within comprehensive MCH services and reduce time‐to‐treatment initiation for the HIV‐infected mother.^56^, ^57^ In Malawi, some ANC providers were trained for counselling but not for HIV testing, necessitating referrals to other providers for testing and resulting in lower rates of HIV testing due to service inefficiencies.^30^ Lack of training was identified as a barrier to delivering an integrated TB‐ PMTCT‐ANC programme in South Africa, where not all healthcare workers were trained in all care aspects, contributing to poor TB case‐finding among pregnant women. Referrals to services provided in different locations also led to inefficiencies and poor uptake.^39^ Introduction of new skills could be done using a step‐wise approach: in Rwanda, a basic initial training was enhanced with additional training, as new services were gradually introduced.^10^ In an integrated ANC delivery model for HIV‐positive infants in Mozambique, staff did not feel that lack of technical knowledge was an impeding factor in their daily work but they appreciated supervisory visits, which provided an opportunity to clarify doubts and receive updated information. Staff also highly valued the regular interest in their work and conditions.^35^

Where guidelines are not available, there is a risk of sub‐optimal service provision. Evidence from multiple country programmes suggest that with integrated malaria services, providers are often not clear about how to deliver intermittent preventative treatment of malaria in pregnancy (IPTp) for specific cases (such as HIV‐positive women) and how to address potential side effects. Given the uncertainty about the care pathways and fear of doing harm, healthcare providers providing integrated malaria services opt for ‘doing nothing’ rather than intervene and risk making an error, leading to lower coverage of IPTp. To overcome this barrier, in Kenya a memo from a senior health official was combined with explanation of the guidelines for follow‐up supervision visits to boost the uptake of IPTp in some districts.^15^

Integrated services, by design, require health workers to assume additional tasks. These additional tasks may lead to increased workload and longer wait times, making health workers reluctant to implement them. In Malawi, clients and providers both voiced concerns about the acceptability of TB screening and treatment as part of ANC, due to a possible rise in workloads and the consequences thereof.^16^ It can be difficult to ask providers to take on additional tasks without extra compensation, unless it is made clear the extra services are now to be considered part of routine ANC services. New responsibilities without adequate compensation are likely to cause poor morale among providers.^16^, ^43^ Some studies suggest that poor morale, lack of motivation and tensions among providers may be higher in large urban health facilities with greater specialisation of services, as different departments and cadres of providers may be more reluctant to share or relinquish authority. By contrast, rural providers who make their own treatment and care decisions have fewer problems adapting the organisation of services to respond better to client needs.^24^ In Kenya, providers at smaller health facilities felt that integration of HIV and ANC services in one location had simplified their work, reducing the need to escort the patient to another section of the facility where the HIV documentation and medications were available.^32^

Integration of new services could potentially motivate health providers, as they feel empowered by being able to deliver more effective and potentially life‐saving services and simultaneously learn how to protect themselves. In Mozambique, for one‐stop integrated MCH services (including ANC) for HIV‐infected infants, staff reported high job satisfaction and a feeling of increased effectiveness, with less time spent on administrative tasks.^35^ In Kenya, providers at integrated sites also noted that they felt more motivated, intellectually challenged, and satisfied with their work as a result of the additional training and new knowledge gained.^32^

In the USAID Horizons Program, healthcare providers in Kenya, Zambia, Zimbabwe and Uganda were trained in multiple service components such as PMCT, rapid HIV tests, VCT, infant feeding, and couples counselling. These training programmes had important positive effects on the attitudes of health workers and on reducing stigma attached to HIV‐positive women.^24^ The motivation from being able to provide integrated PMCT services had encouraged many staff to put extra effort into the care offered to their clients, for example by providing after‐hours counselling and support to mothers living with HIV.^24^

#### Health system constraints

Integration of services does not take place in a vacuum, but is affected by many health system factors.

### Accessibility and utilisation of health services

One of the prerequisites for effective use of ANC as a delivery platform for other health services is the level of ANC coverage. Despite encouraging progress, not all countries have reached ANC coverage targets set nationally or internationally and urban–rural inequities persist.^58^ In Ethiopia, for example, the ACCESS project showed that only 28% of pregnant women attended ANC at least once in their pregnancy, thus presenting a significant barrier to reaching these women with integrated PMTCT services^30^. By contrast, in Thailand the national scaling‐up of PMTCT services in ANC was facilitated by established ANC system structures.^53^ Moreover, not only the uptake but also the timing of ANC visits is important. For instance, although in Fiji nearly all pregnant women have at least one ANC visit, many of them do not attend until late in their pregnancy, significantly reducing the potential success of adding PMTCT services to the ANC encounter.^13^

Organisation of health services also matters. For PMTCT programmes at district hospitals in rural Zimbabwe and Malawi, follow up of mothers and children during the antenatal and postnatal period was hampered by centralisation of activities and poor access to health facilities.^34^, ^50^ To address these challenges, measures were taken to extend PMTCT activities to lower level health facilities, strengthen community outreach programmes and improve geographical access.

### Human resources

Addition of new services to the ANC package places demands on the health workers responsible for their delivery. Unfortunately, in many LMICs lack of human resources across all cadres of health workers is a major bottleneck to service delivery.^59^, ^60^ The health workforce crisis is exacerbated by well‐intended, but ultimately harmful incentive policies that have prompted migration of personnel from reproductive health and family planning programmes to priority programmes, such as those for HIV/AIDS.^61^ An adverse consequence of this internal workforce shift is that health workers who continue to work in ANC and MCH clinics are oftentimes underpaid and overworked. Increases in workload from new tasks, usually accompanied by training, combined with existing shortages and high turn‐over of staff have been highlighted as hurdles to scale‐up of integrated HIV, TB and syphilis services in, among others, Fiji, Uganda, Zambia, South Africa, Swaziland, Mozambique, Uganda and Kenya.^13^, ^16^, ^24^, ^25^, ^35^, ^36^, ^44^, ^45^, ^48^ A study from Tanzania on the impact of integrating and scaling‐up PMTCT on workload showed that integration could be done with existing staff, though this required adjustments in staff productivity and their distribution.^46^

Reluctant to further increase the burden on frontline staff, some countries have sought new approaches to health workforce management, by shifting tasks and engaging new cadres of health workers from the community. In Ethiopia, for example, Health Extension Workers, who were providing a core set of MCH services, were trained also to provide PMTCT measures.^30^ In South Africa the task of providing VCT to pregnant women was given to lay counsellors.^62^ In Cameroon, trained birth attendants provided confidential HIV counselling and testing, allowing for expansion of PMTCT to rural primary health centres.^27^ This resulted in higher acceptance rates by village women, and improved patient education and follow‐up. Although these strategies have improved access to these important services, they risk perpetuating the divide between ANC and other health services and can create confusion about allocation of responsibilities.^21^, ^62^

### Supervision, management and leadership

Clarity on responsibilities for supervision and management of integrated services is vital to ensure quality of care. The ACCESS programme in Kenya found that, although integrated ANC‐PMTCT services had been introduced in over 3000 facilities, supervision was still managed by separate HIV and reproductive health teams, resulting in a lack of information about the overall quality of services.^30^ This misalignment of supervisory roles was subsequently addressed by the joint development of an integrated supervision tool. In South Africa, programmes to integrate TB and PMTCT services also highlighted that, where facilities had separate TB and HIV management guidelines, lack of leadership in the coordination and the supervision of the implementation of joint guidelines were detrimental to the success of integration.^39^

Effective operational management, service delivery, supervision and accountability arrangements are particularly complex in relation to HIV‐related services, where multiple agencies may be involved in financing, planning, service delivery, monitoring and evaluation. All too often each agency has their own systems and resources supporting different interventions, as illustrated by an assessment of the Horizon programme in Zambia, where ‘the Voluntary Counselling and Testing Programme provides VCT, the MTCT Working Group provides ARVs and infant formula, and the District Health Management Team dispenses haemoglobin and iron supplements’.^24^ Challenges to comprehensive integration were reported in South Africa, where PMTCT was administered as a vertical programme, managed by the national HIV coordinating body.^21^, ^62^

### Financial resources

‘Verticalisation’ of health care delivery, resulting in a proliferation of management structures and financing mechanisms, can pose a significant obstacle to service integration.^16^, ^21^ For example, in Tanzania vertically organised remuneration systems for HIV programmes was detrimental to the implementation of integrated PMTCT and MCH services^23^, which required ‘horizontal integration’ of financial and human resources. In other settings, integration of new services, such as malaria treatment and prevention, with ANC have been hampered, as these were not accompanied by sufficient additional funding.^15^

### Information systems

To track performance of newly integrated services, information systems for monitoring and evaluation need to be in place for routine recording and reporting of activities, outputs and outcomes. Although providers at integrated HIV services sites in Kenya observed that integration increased the amount of time spent on recordkeeping, it also led to easier and more accurate reporting of data.^32^ Furthermore, tracking performance of integrated services requires updating of existing ANC supervision checklists and record cards with suitable indicators to ensure capture of relevant data.^24^ For example, under the ACCESS programme in Malawi, integration of PMTCT interventions with existing MNCH services required an update of facility performance indicators and quality standards.^30^

### Procurement and distribution systems

An important health system constraint affecting the success of integrated services is the availability and uninterrupted delivery of commodities and medicines. Unavailability of commodities and irregular supply of essential consumables and drugs were found to be major barriers to uptake of integrated HIV^25^, ^35^, ^44^, syphilis^45^ and malaria services^15^.

### Social and political constraints

Aside from health system constraints, the wider socio‐political context in which the system operates can enable or hinder service integration. In particular, maternal health touches upon fundamental issues of women's rights and religious beliefs that may be contested in different settings. For example, integration of modern contraceptive methods with the ANC package may encounter resistance from faith‐based organisations (FBOs) opposed to the use of such methods, requiring collaboration of all parties to overcome resistance or find creative solutions. In Rwanda, where FBOs provide an important share of health services, the Ministry of Health devised a creative solution by establishing ‘secondary health posts’ adjoining the FBO‐operated facilities, where clients who opt for modern family planning methods may be referred for family planning counselling and to receive health products.^10^

Belief systems and ideologies are often reflected in a country's legal framework and policies, and affect how services are delivered. For instance, integrating HIV screening into the routine prenatal test panel may require a shift from a so‐called ‘opt‐in’ to an ‘opt‐out’ strategy.^13^, ^26^, ^63^, ^64^ However, not all countries have policies that allow opt‐out testing, requiring policy changes to enable integrated service delivery.

Lastly, strong leadership and coordinated efforts of key government agencies, NGOs and donors are critical to the success of uptake and scale up of integrated programmes.^26^, ^51^, ^54^

## Discussion and conclusion

Antenatal care provides an important platform for the delivery of additional health services to women. The rationale for integration is to improve user access to health services across the care continuum to reduce the high levels of ‘loss to follow up’ from first contact with ANC through to postnatal care, help expand coverage and increase the uptake and utilisation of essential services for women and children. Furthermore, integration can reduce programme costs through more synergistic use of human and financial resources.^65^

Benefits of integrating antenatal care with other service can be realised if the services provided, health system design and socio‐political context are carefully considered in the integration process, which requires involvement of all stakeholders. These factors range from those directly concerning the providers and recipients of health services to health system constraints, as well as wider socio‐political environment in which ANC services are delivered.

Provision of multiple services during a single point of contact requires that healthcare providers be sufficiently trained in all aspects of the services concerned to ensure good quality care However, injudicious integration can have undesirable consequences for already constrained and overloaded health systems.^66^ For example, in resource‐constrained health systems, training for integration can take away health staff from frontline services.^67^ Furthermore, provision of multiple services could stretch the limited capacity, leading to longer waiting times and hindering access for women who have to travel far to reach health facilities. In an attempt to reduce workload, providers may reduce the time spent on consultations, thus compromising service quality. Hence integration needs to be carefully planned and implemented.

Health system factors also influence integration of ANC with other services and may require restructuring of existing management, and financing, as well as monitoring and evaluation systems. Hence, the content and complexity of such a service package should be informed by the local health system capacity and the broader contextual factors. While integrated service delivery offers an opportunity for improved access, efficiency and outcomes, in practice implementation has been challenging. To increase chances of success, it is essential to ensure that all parties are involved in the conception, design and implementation of integrated services to create a sense of commitment to and responsibility and ensure ownership.

This study is limited by significant heterogeneity in the studies published as grey literature, as well as by the small number of rigorous studies identified. Notwithstanding these limitations, we draw on reported experiences from the field to highlight several critical factors that enable or hinder in different contexts successful programme integration which could help improve MCH outcomes through holistic provision of quality care, rather than narrow interventions, which is critical for improving for MNCH outcomes.^24^

The review shows a large evidence gap on the factors influencing integration of health services with ANC and the potential benefits. There is a clear need for more rigorously conducted studies, ideally involving comparison between different service delivery models with random allocation. However, additional quasi‐experimental studies, and demonstration projects, complemented by modelling studies, could also provide valuable insights in this area and in particular should help in understanding the role of contextual factors in achieving specific outcomes.

### Disclosure of interests

None declared. Completed disclosure of interests form available to view online as supporting information.

### Details of ethics approval

None required.

### Contribution to authorship

T.dJ. and I.G.U. were responsible for development of the methodology, all stages of data collection and analysis, and reporting. E.A. and J.Z. contributed to data collection, screening of papers and data extraction. R.A. was responsible for the conception of the study and contributed to drafting and finalisation of the manuscript. All authors take responsibility for this study and its findings.

### Funding

This study was supported by a grant from the Bill & Melinda Gates Foundation.

## Supporting information


**Appendix S1.** Medline Search Strategy.
