# Supplementary material for: Barriers and enablers to integrating maternal and child health services to antenatal care in low and middle income countries
Source: BJOG. 2016 Feb 9;123(4):549–57. doi: 10.1111/1471-0528.13898 (PMC4768640; doi:10.1111/1471-0528.13898)
Supplement: Supplementary file 1 — Appendix S1. Medline Search Strategy. [file BJO-123-549-s001.pdf]

Appendix S1. Medline Search Strategy

1. (antenatal or (ANC and pregnan*) or prenatal or pre-natal or perinatal or peri-natal).ti,ab,kf,hw.
2. exp Perinatal Care/ or exp Prenatal Care/
3. 1 or 2
4. exp "Delivery of Health Care, Integrated"/
5. (integrat* adj3 (care or service* or delivery or strateg* or program* or management)).ti,ab,kf,hw.
6. ((deliver* or bring) adj3 (with or within or together)).ti,ab,kf,hw.
7. (integrat* or horizontal or vertical or coordinat* or co-ordinat* or link*).ti,ab,kf,hw.
8. (multi* adj2 (team? or care or service? or clinic?)).ti,ab,kf,hw.
9. (multicare or multiservice? or multiclinic?).ti,ab,kf,hw.
10. (multiskill* or multi skill* or multitask* or multi task*).ti,ab,kf,hw.
11. (continuum or continuity) .ti,ab,kf,hw.
12. or/4-11
13. exp developing countries/
14. exp medically underserved area/
15. (LIC or LICs or MIC or MICs or LMIC or LMICs or LAMIC or LAMICs or LAMI countr* or third world).ti,ab,kf,hw.
16. (low adj3 middle adj3 countr*).ti,ab,kf,hw.
17. ((developing or (less* adj developed) or under developed or underdeveloped or (middle adj income) or (low* adj income) or underserved or under served or deprived or poor* or shortage or rural or remote or nonmetropolitan or transition*) adj (communit* or countr* or nation* or population* or district* or state* or province* or jurisdiction* or region* or area* or territor* or world or economy or economies)).ti,ab,kf,hw.

18. (Afghanistan or Albania or Algeria or Angola or Antigua or Barbuda or Argentina or Armenia or Armenian or Aruba or "Atlantic Islands" or Azerbaijan or Bahrain or Bangladesh or Barbados or Benin or Dahomey or Byelarus or Byelorussian or Belarus or Belorussian or Belorussia or Belize or Bhutan or Bolivia or Bosnia or Herzegovina or Hercegovina or Botswana or Bechuanaland or Kalahari or Brazil or Bulgaria or "Burkina Faso" or "Burkina Fasso" or "Upper Volta" or Burundi or Urundi or Cambodia or "Khmer Republic" or Kampuchea or Cameroon or Cameroons or Cameron or Camerons or "Cape Verde" or "Central African Republic" or Ubangi-Shari or Chad or Chile or China or Colombia or Comoros or "Comoro Islands" or Comores or Mayotte or (Congo not (congo red or crimean-congo)) or Zaire or "Costa Rica" or "Cote d'Ivoire" or "Ivory Coast" or Croatia or Cuba or Cyprus or Czechoslovakia or "Czech Republic" or Slovakia or "Slovak Republic" or "Democratic People's Republic of Korea" or "north korea" or (democratic people* republic adj2 korea) or Djibouti or "French Somaliland" or Dominica or "Dominican Republic" or "East Timor" or "East Timur" or "Timor Leste" or Ecuador or Egypt or "United Arab Republic" or "El Salvador" or Eritrea or Estonia or Ethiopia or Fiji or Gabon or "Gabonese Republic" or Gambia or Gaza or Georgia or Georgian or Ghana or "Gold Coast" or Greece or Grenada or Guatemala or (Guinea not (New Guinea or Guinea Pig* or Guinea Fowl)) or Guam or Guiana or Guyana or Haiti or Honduras or Hungary or India or "Indian Ocean Islands" or Maldives or Indonesia or Iran or Iraq or "Isle of Man" or Jamaica or Jordan or Kazakhstan or Kazakh or Kenya or Kiribati or Korea or Kosovo or Kyrgyzstan or Kirghizia or "Kyrgyz Republic" or Kirghiz or Kirgizstan or "Lao PDR" or Laos or (lao adj1 democratic republic) or Latvia or Lebanon or Lesotho or Basutoland or Liberia or Libya or Lithuania or Macedonia or Madagascar or Melanesia or "Malagasy Republic" or Malaysia or Malaya or Malay or Sabah or Sarawak or Malawi or Nyasaland or Mali or Malta or Marshall Island* or Mauritania or Mauritius or "Agalega Islands" or Mexico or Micronesia or "Middle East" or Moldova or Moldovia or Moldovian or Mongolia or Montenegro or Morocco or Ifni or Mozambique or "Portuguese East Africa" or Myanmar or Myanma or Burma or Namibia or

Nepal or "Netherlands Antilles" or "New Caledonia" or Nicaragua or (Niger not (Aspergillus or Peptococcus or Schizothorax or Cruciferae or Gobius or Lasius or Agelastes or Melanosuchus or radish or Parastromateus or Orius or Apergillus or Parastromateus or Stomoxys)) or Nigeria or "Northern Mariana Islands" or Oman or Muscat or Pakistan or Palau or Palestine or Panama or "Papua New Guinea" or Paraguay or Peru or Philippines or Philipines or Phillipines or Phillippines or Poland or Portugal or "Puerto Rico" or Romania or Rumania or Roumania or Russia or Russian or Rwanda or Ruanda or "Saint Kitts" or "St Kitts" or Nevis or "Saint Lucia" or "St Lucia" or "Saint Vincent" or "St Vincent" or Grenadines or Samoa or "American Samoa" or "independent state of samoa" or "Samoan Islands" or "Navigator Island" or "Navigator Islands" or "Sao Tome" or "Saudi Arabia" or Senegal or Serbia or Montenegro or Seychelles or "Sierra Leone" or Slovenia or "Sri Lanka" or Ceylon or "Solomon Islands" or "South Africa" or Somalia or Sudan or Suriname or Surinam or Swaziland or Syria* or Tajikistan or Tadzhikistan or Tadjikistan or Tadzhik or Tanzania or Zanzibar or Thailand or Togo or "Togolese Republic" or Tonga or Trinidad or Tobago or Tunisia or Turkey or Turkmenistan or Turkmen or Tuvalu or Uganda or Ukraine or Uruguay or USSR or "Soviet Union" or "Union of Soviet Socialist Republics" or Uzbekistan or Uzbek or Vanuatu or "New Hebrides" or Venezuela or Vietnam or "Viet Nam" or "West Bank" or Yemen or Yugoslavia or Zambia or Zimbabwe or Rhodesia).ti,ab,kf,hw.

19. exp China/ or exp Russia/ or exp India/
20. or/13-19
21. 3 and 12 and 20
22. limit 21 to humans
23. 22 not animals/
